# Retention of the Urine

**Published:** 1879-08

**Authors:** 


### RETENTION OF THE URINE.

### Thirty-five Punctures of the Bladder—Recovery.

In the *Revue Medicale de Tolouse*, Dr. Dazet reports a case of treatment for the *retention of urine*, by puncture with the *aspirateur* (exhauster.)

This method of operation, lately introduced into practice, although not so *painless* as in the case cited by our associate, is of very great service. In case of periprostatic congestion, a transient inflammation creates an insurmountable obstacle, one or more punctures corrects the disorder, and the retention ceases without operation upon the canal.

In this case the subject was a man, fifty years of age, attacked with *retention of the urine* succeeding hemorrhoidal congestion.

It being impossible to use the catheter, an opening was made with needle No. 2 of the *aspirateur*. There was a discharge of about four and one-half pints of urine. The next morning a second puncture was made; and, the following evening, a third. From the 27th of July to the 12th of August, this practice was adhered to, morning and evening. Thus there were thirty-five punctures in a space limited to two or three centimetres above the pubis.

The discharge by the urethra commenced after the third puncture, and it was only after the thirty-fifth, that the patient habitually voided the urine in a natural manner. Then he recovered without difficulty.

This result is of great interest in those not rare cases in which it is very difficult, perhaps impossible, to draw off the urine with the usual appliances.—*Journal de Medicine et de Chirurgie Practiques.*

